# A high quality method for hemolymph collection from honeybee larvae

**DOI:** 10.1371/journal.pone.0234637

**Published:** 2020-06-18

**Authors:** Nicole Pavan Butolo, Patricia Azevedo, Luciano Delmondes de Alencar, Caio E. C. Domingues, Lucas Miotelo, Osmar Malaspina, Roberta Cornélio Ferreira Nocelli

**Affiliations:** 1 Centro de Estudos de Insetos Sociais–CEIS, Instituto de Biociências–Programa de Pós Graduação em Biologia Celular e Molecular, Universidade Estadual Paulista ‘Júlio de Mesquita Filho’ (UNESP-SP), Rio Claro, SP, Brazil; 2 Grupo de Genética e Genômica da Conservação, Instituto de Biologia–Programa de Pós Graduação em Genética e Biologia Molecular, Universidade Estadual de Campinas (UNICAMP-SP), Campinas, SP, Brazil; 3 Departamento de Ciências da Natureza, Matemática e Educação, Centro de Ciências Agrárias, Universidade Federal de São Carlos (UFSCar-SP), Araras, SP, Brazil; University of North Carolina at Greensboro, UNITED STATES

## Abstract

The drastic decline of bees is associated with several factors, including the immune system suppression due to the increased exposure to pesticides. A widely used method to evaluate these effects on these insects' immune systems is the counting of circulating hemocytes in the hemolymph. However, the extraction of hemolymph from larvae is quite difficult, and the collected material is frequently contaminated with other tissues and gastrointestinal fluids, which complicates counting. Therefore, the present work established a high quality and easily reproducible method of extracting hemolymph from honeybee larvae *(Apis mellifera)*, the extraction with ophthalmic scissors. Extraction methods with the following tools also were tested: 30G needle, fine-tipped forceps, hypodermic syringe, and capillaries tubes. The hemolymph was obtained via an incision on the larvae’s right side for all methods, except for the extraction with ophthalmic scissors, in which the hemolymph was extracted from the head region. To assess the purity of the collected material, turbidity analyses of the samples using a turbidimeter were proposed, tested, and evaluated. The results showed that the use of ophthalmic scissors provided the clearest samples and was free from contamination. A reference range between 22,432.35 and 24,504.87 NTU (nephelometric turbidity units) was established, in which the collected samples may be considered of high quality and free from contamination.

## Introduction

The honeybee *Apis mellifera* has been used worldwide as a model organism in several studies because it is a species with widely known biology, has a wide geographical distribution, is easily managed and maintained in laboratories, a great indicator of environmental quality and the most frequent floral visitor of agricultural crops [1, 2, 3]. Therefore, several studies in different areas of science use this species, including studies that evaluate the impact of pesticides [4, 5, 6, 7], host-parasite interactions [8, 9, 10, 11, 12, 13], behavioral tests [14, 15, 16] and at the molecular level genomic, transcriptomic and epigenetic patterns [17, 18, 19, 20].

Despite its importance, the bees’s population decline have increased dramatically, mainly due to the loss and fragmentation of habitats, fires, deforestation, increased parasites and diseases, climate change, and the increased exposure to pesticides [2, 21, 22, 23]. Pesticides act on different systems in these insects, and the first defense system of an organism, the immune system, is extremely affected. Honeybees have a reduced set of the immune genes [24].

If a foreign agent or molecule overcomes the first obstacles of the insects’ innate immune system and physical barriers, humoral and cellular reactions are the next mechanisms activated [25, 26, 27]. The humoral reaction consists of the hemolymph melanization due to oxidative processes and the action of proteins, and the cellular reactions consist in the action of defense cells, i.e., hemocytes, which perform various functions, such as phagocytosis, nodulation, encapsulation, enzymes secretion, hemolymph coagulation, and nutrient transport [25, 28, 29].

The honeybee's hemolymph is composed of several proteins and different hemocyte types [30, 31, 32, 33, 34, 35]. Hemocyte concentrations vary according to the developmental stage and the honeybee caste and are generally found in fifth instar larvae at a hemolymph concentration of 10,000 hemocytes/mL [36], 21,000 hemocytes/mL in winter workers and drones [37] and 1,000 to 4,000 hemocytes/mL in queens [25]. Therefore, one method that is widely used to assess different impacts on the bees' immune system is the counting of circulating hemocytes in the hemolymph.

Research has found that in colonies exposed to the pesticide thiamethoxam exhibit an increase in the number of circulating hemocytes in the hemolymph of *A*. *mellifera* adult honeybees [38, 39], such as in the honeybee *Apis dorsata* F. exposed to thiacloprid [40], as an immediate immune system response to defend the organism. In contrast, experiments on prolonged exposure of adult honeybees and queens to thiacloprid, imidacloprid and clothianidin indicated a reduction in hemocytes number and the oxidative activity of the hemolymph; these variations in the hemocytes number occur due to the honeybees’ age, caste, insecticide concentration and exposure time [26, 41]. Parasitism by *Varroa* mites reduces the hemocyte number, proteins, and hemolymph enzymes [42, 43]. Researches related to immune system activities of *A*. *mellifera* larvae honeybees exposes to pesticides may result in overexpression of detoxification enzymes [44] and susceptibility to viruses [45]. This scenario is more damaging to Brazilian native stingless bees *Melipona scutellaris*, which showed more sensitivity to the insecticide dimethoate [46].

Even though, studies evaluating the impact on the bees' immune system are important and enlightening, these studies are difficult or avoided due to the difficulty of extracting quality material without contamination. If not properly isolated, the extracted material may be contaminated by other tissues, which hampers cellular and molecular studies. This difficulty is even greater in the larval stage because the biological material manipulation must be meticulous to avoid damage to the tissues and the consequent material loss, and larvae have a large amount of fat body tissue, which makes it difficult to adequately isolate the hemolymph [47].

The different techniques for the hemolymph extraction in the larval stage described in the literature include puncture of the abdomen with a fine capillary tube [48], making a small incision in the larvae’s second third lateral [35], cuticle puncture with the aid of fine-tipped forceps [27], cuticle puncture with a fine hypodermic needle (30-gauge) [49, 50], or piercing of the lateral cuticle with a 0.45 mm diameter pin [51]. However, none of these techniques has proved to be effective to collect the material with suitable purity for laboratory tests. Therefore, the present study standardized a new method of hemolymph extraction from *A*. *mellifera* honeybee larvae that was free of contamination by fat body tissue or gastrointestinal fluids. We also compared all methods of hemolymph extractions from larvae to demonstrate the difficulties of each technique and assess the viability and purity of the collected material.

## Materials and methods

Research on invertebrates does not require animal ethics approval in Brazil.

### Biological materials

*A*. *mellifera* honeycombs containing fifth instar larvae were collected from three non-parental colonies that were free of symptomatic diseases and located in the apiary of the Departamento de Biologia, Instituto de Biociências of Universidade Estadual Paulista (UNESP), Rio Claro, São Paulo, Brazil (22° 23' 48.1" S; 47° 32' 33.1" W). Subsequently, the combs were placed in a biochemical oxygen demand (BOD) incubator at 34°C (± 2) and humidity of 80% (± 5%) at the Laboratório de Ecotoxicologia e Conservação de Abelhas of the Centro de Estudos de Insetos Sociais from UNESP.

### Materials used in the extraction

Different materials were used for the different extraction techniques tested: 11 cm fine-tipped forceps (stainless steel), capillaries tubes (0.22 mm and 0.8–1 mm), 30G needle, 1 mL hypodermic syringe and 9 cm ophthalmic scissors (stainless steel).

It is important to point out that sterile samples extractions must be performed in a laminar flow chamber, with the material previously sterilized by ultraviolet light for 25 minutes. Besides, it is recommended that the larvae are disinfected by immersion in 70% alcohol for 1 minute, washing by immersion in autoclaved water for 1 minute (2x) and let them dry on autoclaved filter paper for approximately 2 minutes [52, 53], or by raising larvae *in vitro* in a sterile environment.

### A new method of hemolymph extraction

#### Ophthalmic scissors

The hemolymph larvae extraction using 9 cm ophthalmic scissors (Argos^®^, Belo Horizonte, Minas Gerais, Brazil, model 4004) was tested, and, unlike the other techniques described, the hemolymph was not removed via lateral puncture, but through a small incision in the head region. This technique is not described in the literature and is being proposed as a new method in this work.

**Fig 1:**
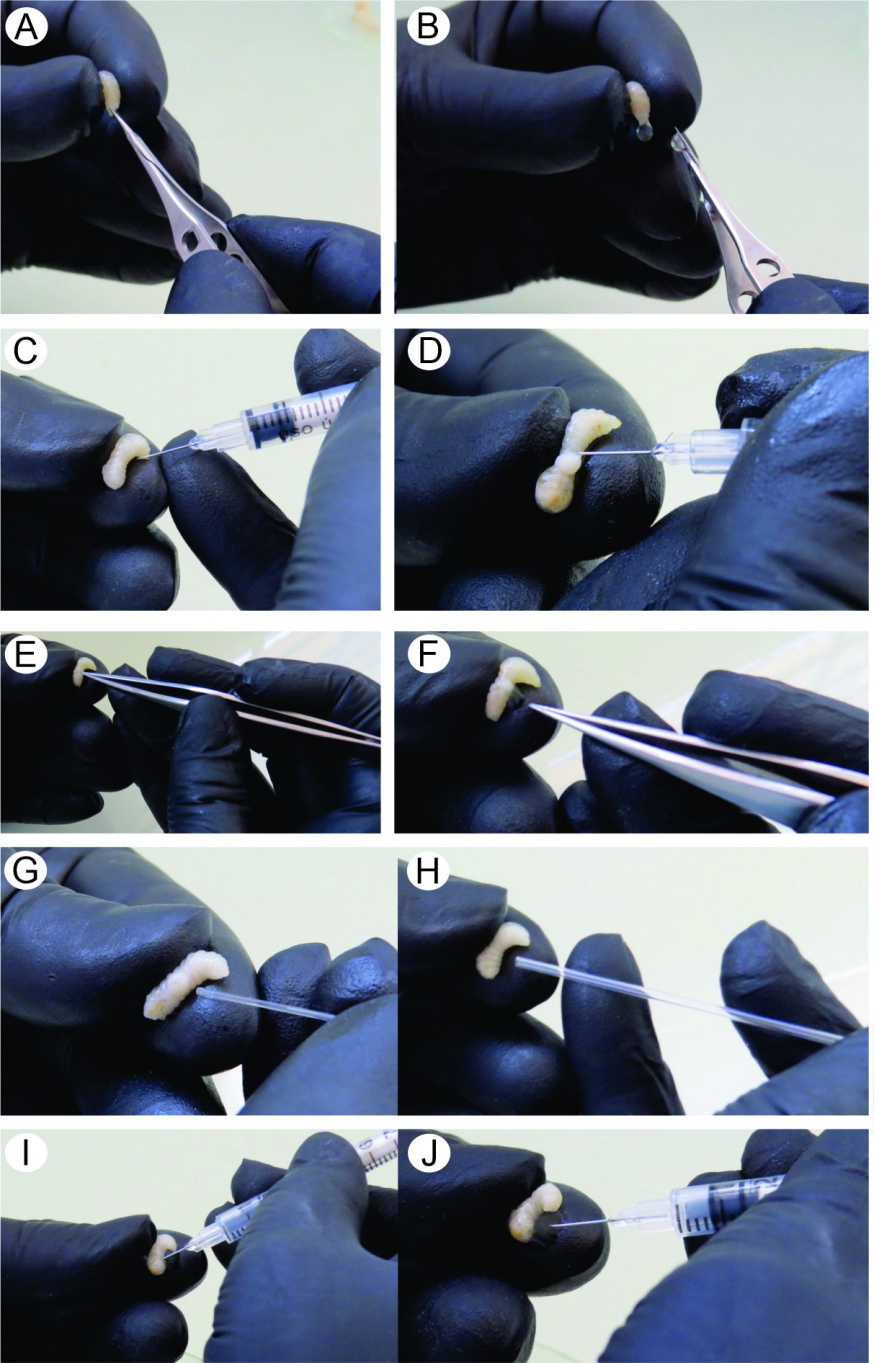
Different techniques for extracting hemolymph from the fifth instar larvae of *A*. *mellifera*. (A and B) Hemolymph extraction from larvae using ophthalmic scissors; (C and D) Hemolymph extraction from larvae using a 30G needle; (E and F) Hemolymph extraction from larvae using fine-tipped forceps; (G) Hemolymph extraction from larvae using a 0.22 mm capillary tube; (H) Hemolymph extraction from larvae using a 0.8–1.1 mm capillary tube; (I and J) Hemolymph extraction from larvae using a hypodermic syringe.

**Fig 2:**
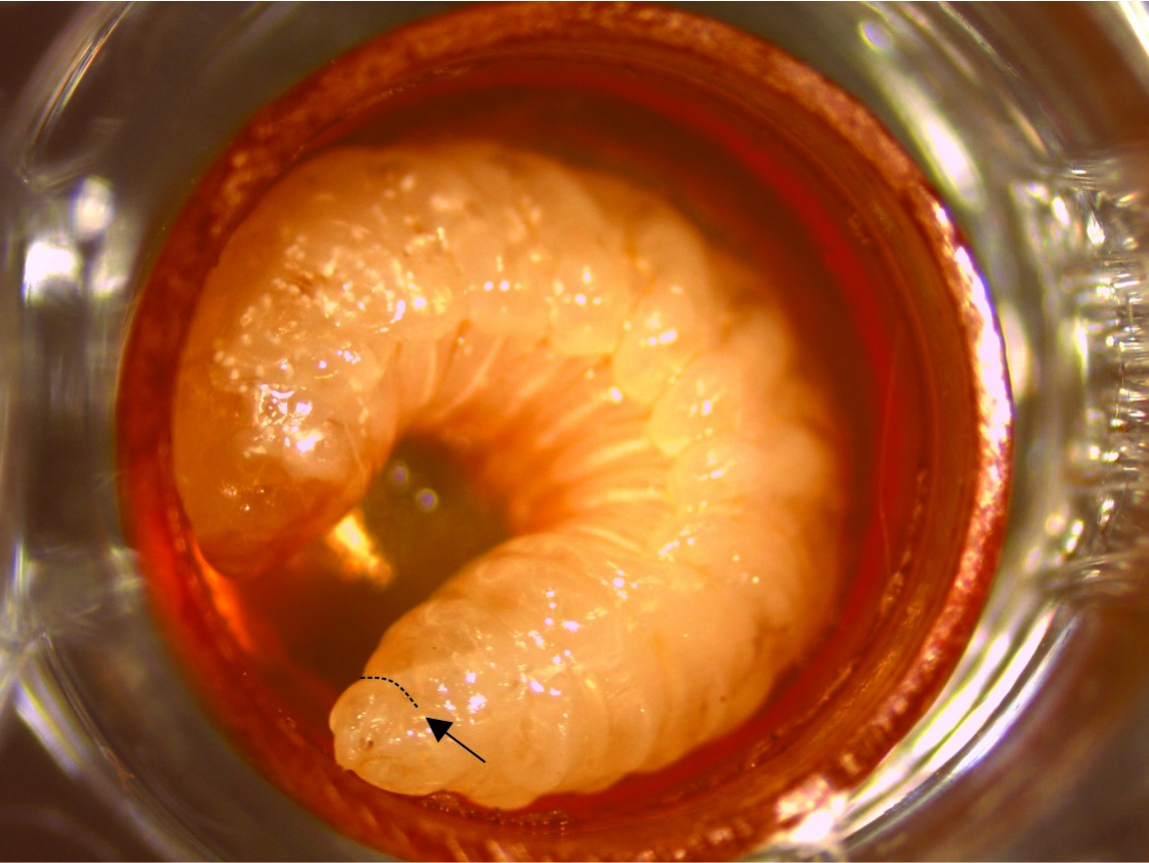
Scheme indicating the exact incision in the ventral region of fifth instar *A*. *mellifera* larva head. The arrows indicate the ventral region and the dashed indicate the incision site.

First, the larvae were immobilized holding by the index and thumb fingers using the non-dominant researcher’s hand (Fig 1A and 1B). Subsequently, a small incision was made in the cuticle corresponding to the larvae’s head region (Fig 2) and light pressure was applied to the body to extravasate the hemolymph. The liquid was collected using an automatic micropipette and placed in a polypropylene microtube for further analysis. The puncture may also be performed with fine-tipped forceps (Argos^®^, Belo Horizonte, Minas Gerais, Brasil, model 1040). However, the scissors provide greater precision and do not cause tissue damage, which guarantees higher sample quality and faster extraction.

### Other tested methods of hemolymph extraction techniques

#### 30G needle

The technique of extracting hemolymph from larvae using a 30G needle [49, 50] was performed with the aid of a 1 mL hypodermic syringe coupled to a thin hypodermic needle. The larvae were immobilized using the same immobilization technique described in the first extraction item to expose the side of the larvae (usually the right side), and the needle was inserted into the lateral cuticle. Light pressure was applied to the larvae's body so that the extraction contents overflowed. The hemolymph was collected from the punctured orifice with the aid of an automatic micropipette and placed in polypropylene microtubes for further analyses (Fig 1C and 1D).

#### Fine-tipped forceps

For the extraction of hemolymph using 11 cm fine-tipped tweezers (Argos^®^, Belo Horizonte, Minas Gerais, Brazil, model 1040), the larvae were immobilized using the same immobilization technique described in the first extraction item to expose the side of the larvae and, the lateral cuticle was punctured with the aid of a fine-tipped forceps [27]. Light pressure was applied to allow the hemolymph to overflow through the open hole, and the exposed content was collected using an automatic micropipette and placed in a polypropylene microtube for further analysis (Fig 1E and 1F).

#### Capillary tube

The extraction of hemolymph using capillaries tubes was tested as proposed by Randolt et al. [48]. The same immobilization technique was used as described in the first extraction item, and the capillary tube was inserted into the exposed side until the cuticle was broken. The internal content was collected via pressure difference and removed with the aid of a rubber bulb. Two capillaries tubes sizes were used, 0.22 mm (Drummond Microcaps^®^, Drummond Microcaps^®^, Broomall, Pennsylvania, United States) and 0.8–1.1 mm (Kimax Capillary^®^, Mainz, Rhineland-Palatinate, Germany) (Fig 1G and 1H).

#### Syringe

The extraction of hemolymph from larvae using a syringe was tested to assess its viability and ease. This technique is not described in the literature. The same immobilization technique was used as described in the first extraction item to expose the larvae side, and a 30G needle attached to a hypodermic syringe was inserted into the larval side cuticle. The hemolymph was aspirated with the syringe and placed in polypropylene microtubes for further analysis (Fig 1I and 1J).

### Feasibility assessment of different extraction techniques

#### Light microscopy

After extraction, the hemolymph samples obtained from the different extraction techniques were evaluated and photo-documented under a light microscope (OLYMPUS^®^, model BX51). The samples were stained with 1:1 methylene blue (5 μL of methylene blue and 5 μL of the sample) and observed in a Neubauer chamber (Marienfield^®^, Lauda-Konigshofen, Germany).

Photomicrographs were acquired using a digital camera (OLYMPUS^®^, Tokyo, Japan, model DP-71) adapted to a bright-field light microscope and a computer (Dell^®^, Round Rock, Texas, United States). DP Controller^®^ (Round Rock, Texas, United States) software was used to acquire the images.

#### Protein content measurement—Bradford method

The Bradford method was used to determine the concentration of proteins present in the samples extracted from each of the techniques [54]. Initially, a calibration curve was made with a previously known concentration of bovine serum albumin (BSA) standard. Subsequently, Coomassie Brilliant Blue G-250 (BioRad^®^, Santo Amaro, Sao Paulo, Brazil) dye (0.01%) was added, and the plate was read on a plate reader (Hexis^®^, Judiai, Sao Paulo, Brazil, model Versamax) at 595 nm.

For analysis of hemolymph samples from the different extraction techniques tested, 10 μL of hemolymph was diluted in 990 μL of distilled water (100x dilution). Five microliters of the diluted hemolymph samples were placed in a 96-well plate with 195 μL of the protein reagent. After 10 minutes of the reaction at room temperature, the plate was read on a plate reader at 595 nm. The values obtained were analyzed and compared with the calibration curve to establish the concentration of proteins present in the samples (S1 and S2 Datas).

### Turbidity degree quantification

#### Turbidimeter method

The samples’ turbidity analysis was proposed because of the different extraction techniques produced hemolymph with different properties due to the presence of solids and milky samples. A turbidimeter (Hach^®^, Loveland, Colorado, United States, model 2100Q) was calibrated according to the manufacturer's instructions. The formazine standards were used in different concentrations of NTU (nephelometric turbidity units): 20 NTU, 100 NTU, and 800 NTU. After, 10 NTU standard was used to confirm the calibration. Readings of the hemolymph samples extracted were performed according to the different techniques mentioned. For the samples to be within the reading range of the device, it was diluted 2,500 times:10 μL of hemolymph (extracted from one larva) was added to 24,990 μL of distilled water, for a total volume of 25 mL, which was necessary for reading on the turbidimeter. The values obtained in NTU were multiplied by the dilution factor to obtain the real value of turbidity in NTU. Thirty readings (30 larvae) of hemolymphs were taken for each of the different techniques tested. To confirm the robustness and reproducibility of the tests, the extractions from the different turbidimeter techniques and readings were repeated on three different days totalizing 90 readings for each technique. All data information is available in the Supporting Information (S3 Data).

### Statistical analyses

The turbidity and protein quantification analyses were performed for the different extraction techniques were compared using the R (R Core Team) program. First, the data obtained were subjected to ANOVA, and the normality of the data was verified using the Shapiro-Wilk test. The results were subjected to Tukey’s test at a 1% probability of error (p<0.01). The reference values established for the turbidity analysis (turbidimeter method) were also generated using the R program (R Core Team).

## Results

### Feasibility assessment of different extraction techniques

#### Light microscopy

**Fig 3:**
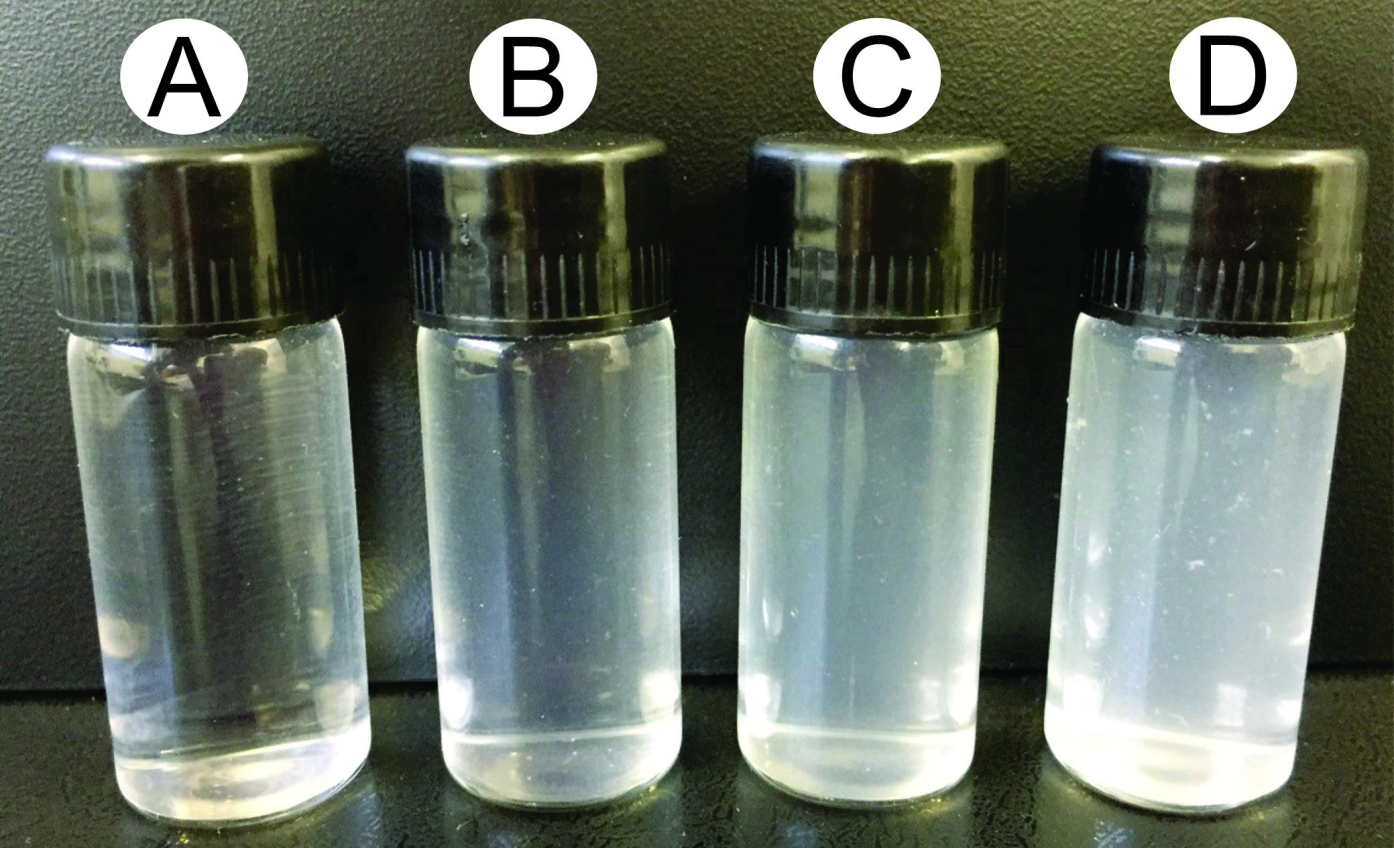
Turbidity observed in the different hemolymph samples of larvae in the fifth instar extracted using the different proposed extraction techniques. (A) Hemolymph extraction using ophthalmic scissors; (B) Hemolymph extraction using a hypodermic syringe; (C) Hemolymph extraction using a 30G needle; (D) Hemolymph extraction using fine-tipped forceps. The four different samples were diluted 2,500x for viewing under the light microscope.

After the hemolymph extraction from the *A*. *mellifera* fifth instar larvae using the different techniques proposed, high turbidity was observed (Fig 3) in most of the samples, which invalidates their use in the analysis because the desirable physical properties of the hemolymph are clarity and translucency, as described in the literature [25]. These variations in turbidity were evaluated via visualization under a light microscope and Neubauer chamber counting to confirm the different cell types present in the samples. Notably, the masses responsible for making the samples cloudy and milky consisted of larvae’s fat body parts [35].

**Fig 4:**
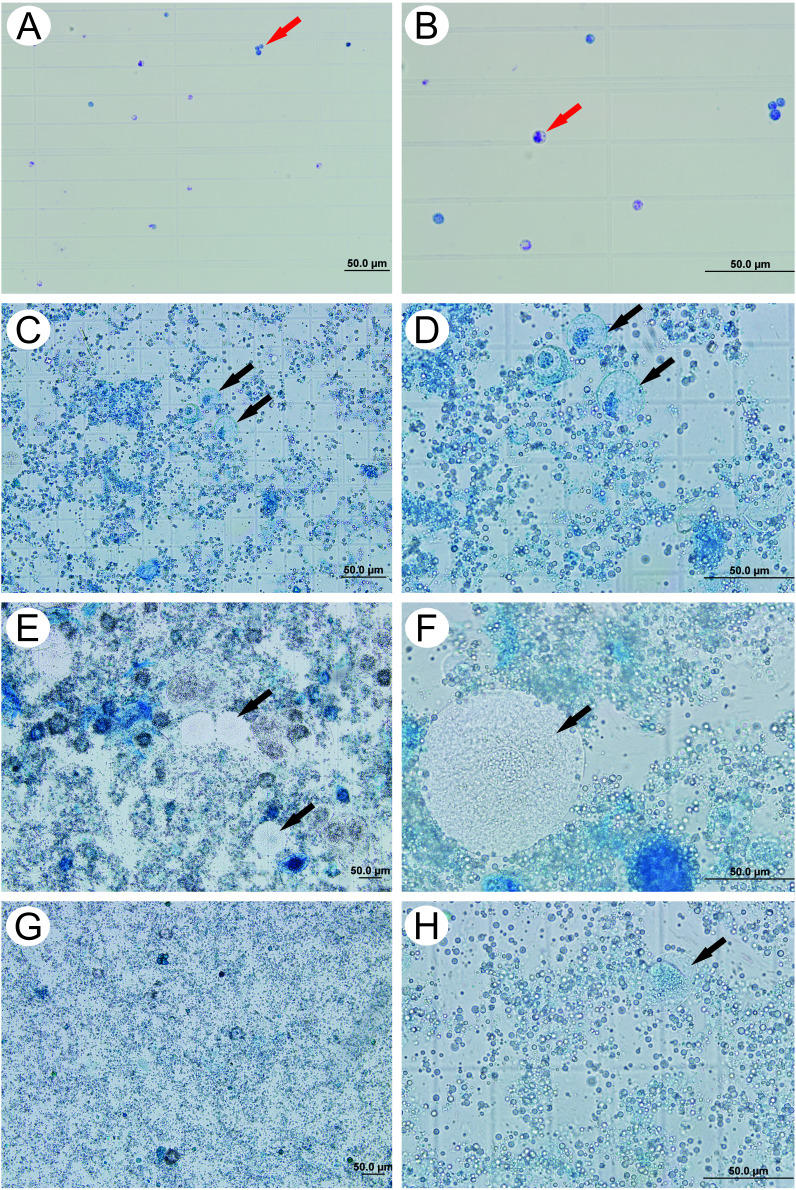
Photomicrographs of Neubauer chambers containing hemolymph samples from fifth instar larvae of *A*. *mellifera* obtained using different extraction techniques and stained with methylene blue 0.2%. (A) Ophthalmic scissors extraction at 200x magnification; (B) Ophthalmic scissors extraction at 400x magnification (C) 30-G needle extraction at 200x magnification; (D) 30G needle extraction at 400x magnification; (E) Fine-tipped forceps extraction at 100x magnification; (F) Fine-tipped forceps extraction at 400x magnification; (G) Hypodermic syringe extraction at 100x magnification; (H) Hypodermic syringe extraction at 400x magnification. Black arrows indicate fat body cells. Red arrows indicate hemocytes.

The photomicrographs showed that the hemolymph obtained by the extraction technique with fine-tipped forceps (Fig 4E and 4F) had more particulate materials compared to the samples extracted with a 30G needle (Fig 4C and 4D) and hypodermic syringe (Fig 4G and 4H). The photomicrographs obtained from the extraction technique with the aid of the syringe allowed us to infer that the particulate material in the sample was more dispersed, which makes it difficult to visualize. The suction of the syringe during the hemolymph extraction may have homogenized the sample and promoted cell lysis and leading to a mixing of the materials. Finally, the hemolymph samples obtained from the ophthalmic scissors extraction were the most viable and least visually cloudy because it was free of cells from other tissues and contaminating materials, and these samples were the easiest to evaluate in the photomicrographs obtained (Fig 4A and 4B). The extraction performed using the capillary tube technique was not feasible because the capillaries clogged right after perforation of the larval cuticle, which made it impossible to observe the samples taken using this technique (S1 and S2 Videos).

#### Protein content measurement—Bradford method

**Fig 5:**
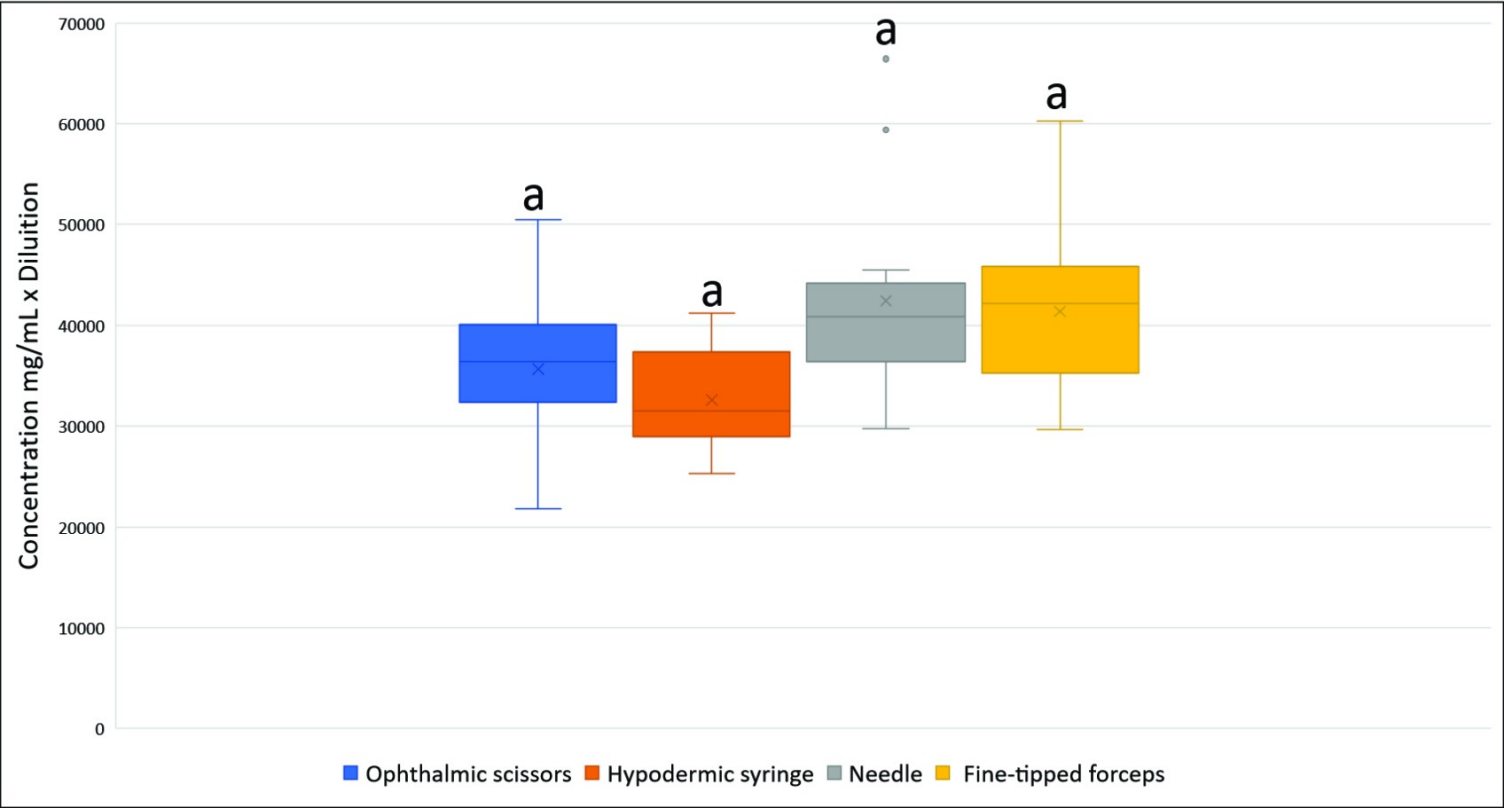
Boxplots comparing protein concentrations in mg/mL (Bradford method) of hemolymph samples from fifth instar larvae of *A*. *mellifera* obtained from different extraction techniques. Boxplots followed by the same letter do not differ by Tukey's test at 5% probability (p<0.05).

The determination of protein concentration in the samples extracted using the different proposed techniques did not show significant differences between treatments at the level of 5% probability of error (p<0.05) (Fig 5). According to the initial hypothesis, the high amount of different cell types and different tissues and/or contamination increased the protein content of cloudy samples compared to clear samples. However, the proteins’ quantification was not sufficient to produce differences in the samples’ groups obtained from the different extraction techniques, and therefore, the initial hypothesis was refuted.

### Turbidity degree quantification

#### Turbidimeter method

All samples' turbidity readings from the different *A*. *mellifera* larvae’s hemolymph extraction were significantly different at the level of significance at 1% probability of error (p<0.01), but there were no differences concerning the day in which the analyses were performed (Table 1).

**Table 1:** Values of the turbidity measurements of the samples in NTU. Comparison between the three days of sample collection tested in a turbidimeter, between the four different techniques used for the extraction of hemolymph. Means followed by the same letter in the column did not differ statistically by Tukey’s test at a 1% probability of error.

Techniques	NTU day 1	NTU day 2	NTU day 3
Ophthalmic scissors	23,992 d	23,392 d	23,600 d
Fine-tipped forceps	176,542 a	179,217 a	176,675 a
Needle 30G	120,200 b	122,000 b	120,950 b
Hypodermic syringe	78,683 c	80,267 c	78,475 c
CV %	6.1876	6.1449	5.2375
HSD	4158.40	4188.95	3522.41
p Value	<0.01	<0.01	<0.01

The hemolymph samples from larvae extracted with fine-tipped forceps showed an average of approximately 177,44 NTU. Hemolymph samples extracted from larvae using the 30G needle showed approximate average turbidity of 121,050 NTU, and hemolymph samples from larvae extracted using the syringe showed average turbidity of approximately 79,141 NTU. The hemolymph extraction technique from larvae using scissors showed an average turbidity of 23,661 NTU, which was the least turbid and had the least amount of suspended solid material.

**Fig 6:**
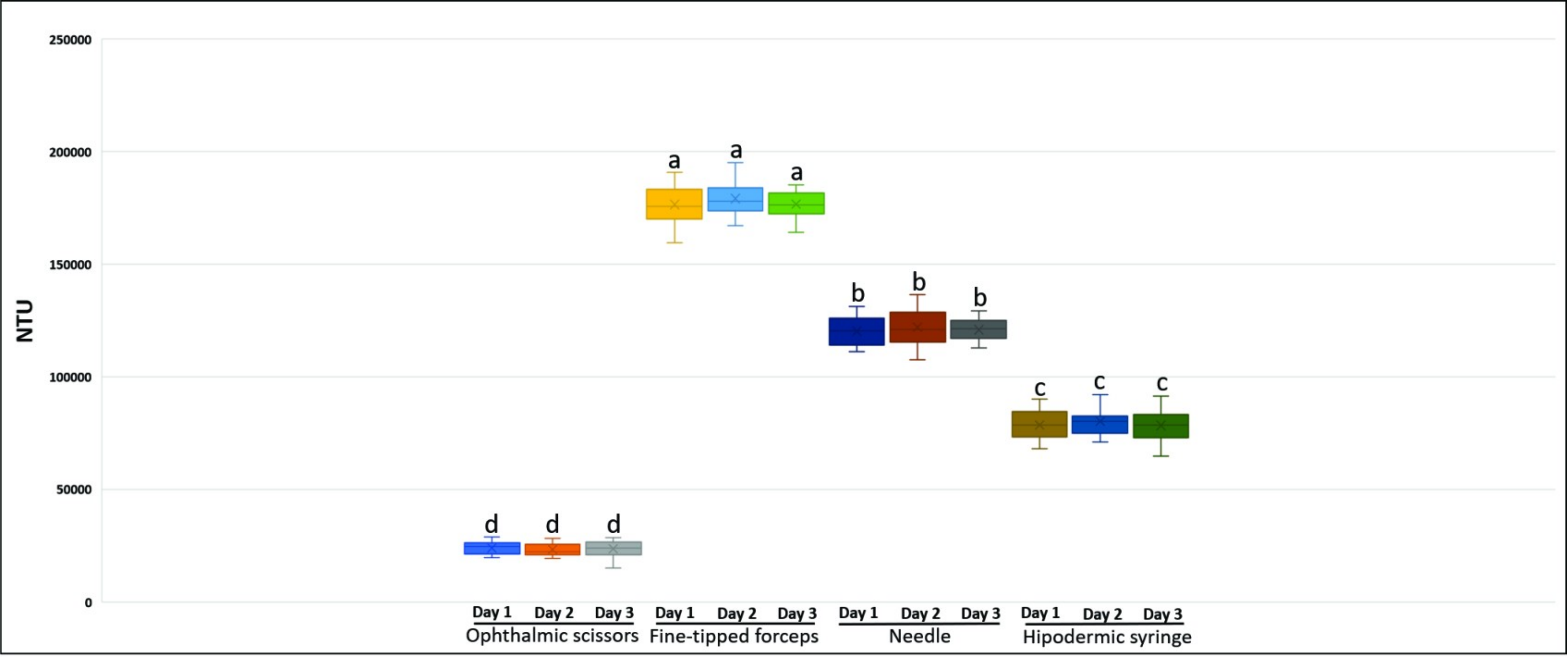
Boxplots comparing the hemolymph samples’ turbidity extracted from the different methods of extraction of hemolymph from fifth instar larvae *A*. *mellifera* tested on three different days. Boxplots followed by the same letter do not differ by Tukey's test at a 1% probability of error (p<0.01).

The tests were repeated twice more to confirm the robustness and reproducibility. Statistical analyses revealed that the turbidity readings of the samples extracted with the ophthalmic scissors on three different days (experimental replication) were not significantly different at the level of significance at 1% probability of error (p<0.01) (Fig 6), which confirms the replicability of the method.

#### Determination of the reference value

To establish a quick and accessible test to verify the purity of the hemolymph extracted from fifth instar larvae, a confidence interval (CI) was calculated based on the turbidity values obtained from the hemolymph samples extracted using ophthalmic scissors because these samples were less cloudy and contaminated with other tissues according to the photomicrographs. However, reference intervals do not exist for the type of reading proposed, and few studies include reference values in invertebrates. Therefore, methods described for vertebrates [55] were used, which were very similar for invertebrates [56], to obtain a reference value for use as a parameter for assessing the purity of hemolymph.

The reference value was determined from the confidence interval calculated based on the NTU values obtained from the readings on the turbidimeter for the samples extracted with ophthalmic scissors. The average NTU value was 23,468 ± 1,036 (standard deviation). Therefore, the confidence interval for the reading given in NTU as an acceptably clear and pure hemolymph sample was 22,432 to 24,504.

## Discussion

The different methods of analyses of extracting hemolymph from fifth instar larvae *A*. *mellifera* demonstrated that the most effective method was the extraction with ophthalmic scissors, making a small incision in the head region. This technique was faster and less susceptible to contamination because contact with the researcher's hand and the mixture of other larval tissues with the collected hemolymph was more unlikely. Therefore, the samples showed significantly higher purity and quality compared to the other extraction methods.

The difficulty of handling and puncturing the *A*. *mellifera* larvae is primarily due to the stage of development of the insect. The cuticle is not fully sclerotized in the fifth larval instar, which makes its manipulation difficult, and organs and systems are not fully developed and defined [57]. This stage is also characterized by a more developed fat body tissue, which occupies the entire parietal and perivisceral space [25, 57], and an increase in the size or number of some tissue cells’, which also influences differentiation and distribution [44, 58]. In the pre-pupa stage, the amount of fat body is drastically reduced, and the dissociation of this tissue leaves a greater amount in the abdomen, which facilitates the hemolymph collection in adult bees because the fat body is primarily concentrated in only one region, and tissue mixing is avoided [25, 59, 60].

As a larval storage tissue [25], the fat body is also responsible for the synthesis of proteins in the hemolymph [61]. Therefore, a high amount of cells should be directly related to a high amount of proteins in the samples. However, protein quantification using the Bradford method [54] did not show significant differences between the different extraction methods. According to Kruger [62], lipids interact with the proteins present in the sample and prevent the proteins from reacting with the dye, which makes it impossible to correctly quantify proteins, and the interaction of Bradford reagents with lipids can also cause turbidity in the sample, which makes proper reading impossible. Therefore, it was concluded that the samples had a high content of lipids, which are essential for the development of the larvae [25, 57].

The physical characteristics of the samples made it possible to evaluate their purity because the different extraction techniques tested showed different intensities of turbidity, i.e., the more turbid the sample, the greater the amounts of 'masses' observed. These masses were later identified as fat body cells with the naked eye and under a light microscope [25, 63, 64, 65, 66]. Therefore, due to the impossibility of quantifying proteins to classify the purity of the hemolymph samples extracted from the larvae, we opted for the physical characterization of turbidity using a turbidimeter. The turbidimeter, or optical back-mirror sensor, measures the amount of suspended solid material from the emission of light beams at an angle of 90° through the sample being analyzed. These beams are deflected when they reach solid particles in the samples, which causes the photodetector to detect different intensities of light that are converted into a photocurrent, which is finally measured in NTU [67, 68].

Despite the feasibility and simplicity of the method, there are no reports in the literature about protocols for reading turbidity from liquid body samples to assess purity. The methods described in the literature for the collection of hemolymph from larvae are not viable for producing pure samples because these methods characterize the hemolymph samples from larvae as nebulous and whitish and link these characteristics to cell clusters. However, the clusters are characteristic of fat body tissue, which makes cell counting and characterization inaccurate because this tissue has different cell types than the cells found in hemolymph [35, 69].

Therefore, the method of characterizing hemolymph by assessing its turbidity was a simple, inexpensive, and viable technique for measuring the purity of body fluids, and it is also easily reproducible in other laboratories. Our results and statistical analyses showed that it was possible to determine an ideal reference value to classify the hemolymph as pure based on the turbidity of hemolymph samples extracted from *A*. *mellifera* larvae [55, 56, 57]. The established reference value will serve as a standard for future tests of this type of material that depend on uncontaminated samples.

## Supporting information

S1 Data Bradford descriptive statistics. (PDF)

S2 Data Bradford raw data. (XLSX)

S3 Data Turbidity descriptive statistics. (PDF)

S1 Video Capillary tube extraction (0.22 mm). (MP4)

S2 Video Capillary tube extraction (0.8–1.1 mm). (MP4)
