# Supplementary material for: A high quality method for hemolymph collection from honeybee larvae
Source: PLoS One. 2020 Jun 18;15(6):e0234637. doi: 10.1371/journal.pone.0234637 (PMC7302910; doi:10.1371/journal.pone.0234637)
Supplement: S1 Data — (PDF) [file pone.0234637.s001.pdf]

Bradford analyses

Summary

Groups	Count	Sum	Average	Variance
Column 1	15	432833.3	28855.55556	7.42E^08
Column 2	15	412666.7	27511.11111	4.15E^08
Column 3	15	620333.3	41355.55556	1.04E^08
Column 4	15	617583.3	41172.22222	59085780

ANOVA

Source of variation	SS	df	MS	F	P-value	F crit
Between Groups	2.58E^09	3	860107523.1	2.605489	0.060694	2.769431
Within Groups	1.85E^10	56	330113657.4			
Total	2.11E^10	59				

Completely randomized design

Data:

Treatments	1ª Rep.	2ª Rep.	3ª Rep.	4ª Rep.	5ª Rep.
Treatment 1	-66250.000	21833.0000	34750.0000	36583.0000	33167.0000
Treatment 2	-43917.000	25333.0000	32833.0000	37250.0000	37750.0000
Treatment 3	66417.0000	34083.0000	59417.0000	26500.0000	40583.0000
Treatment 4	33917.0000	46250.0000	45083.0000	45833.0000	39250.0000

Treatments	6ª Rep.	7ª Rep.	8ª Rep.	9ª Rep.	10ª Rep.
Treatment 1	35417.0000	50500.0000	39000.0000	36167.0000	29917.0000
Treatment 2	40917.0000	41167.0000	30250.0000	29917.0000	28583.0000
Treatment 3	34167.0000	41500.0000	29750.0000	45500.0000	43750.0000
Treatment 4	40333.0000	38500.0000	44083.0000	44500.0000	38250.0000

Treatments	11ª Rep.	12ª Rep.	13ª Rep.	14ª Rep.	15ª Rep.
Treatment 1	40000.0000	40167.0000	23250.0000	41333.0000	37000.0000

Treatment 2	29083.0000	34333.0000	25500.0000	29667.0000	34000.0000
Treatment 3	41333.0000	37167.0000	41083.0000	39333.0000	39750.0000
Treatment 4	60250.0000	35667.0000	29667.0000	30083.0000	45917.0000

Descriptive treatments statistics :

Treatments	Average	Variance	SD	SEM
Treatment 1	28855.6000	741574703	27231.8693	7031.23841
Treatment 2	27511.0667	415369254	20380.6097	5262.25081
Treatment 3	41355.5333	104432032	10219.1992	2638.58588
Treatment 4	41172.2000	59084872.0	7686.66846	1984.68926

Analysis of variance.

	df	SS	MQ	F	P
Treatments	3	2580315641.7	860105213.91	2.61NS	0.0607
Residual	56	18486452045	330115215.08	-	-
Total	59	21066767686	-	-	-

Average......................: 34723.600
SD..............................: 18169.073
standard error ....: 4691.2345
coefficient of variation: 52.324854

Teste de Tukey

Treatment	Variable 1	
Treatment 3	41355.533	a
Treatment 4	41172.200	a
Treatment 1	28855.600	a
Treatment 2	27511.067	a

Significant difference (5%) = 17567.1630
