# Supplementary material for: A high quality method for hemolymph collection from honeybee larvae
Source: PLoS One. 2020 Jun 18;15(6):e0234637. doi: 10.1371/journal.pone.0234637 (PMC7302910; doi:10.1371/journal.pone.0234637)
Supplement: S3 Data — (PDF) [file pone.0234637.s003.pdf]

Turbidity analyses

Summary

Groups	Count	Sum	Average	Variance
Column 1	30	719750	23991.67	7351221
Column 2	30	701750	23391.67	7119325
Column 3	30	708000	23600	12640517
Column 4	30	5296250	176541.7	64077945
Column 5	30	5376500	179216.7	47291954
Column 6	30	5300250	176675	28707543
Column 7	30	3606000	120200	40928448
Column 8	30	3660000	122000	67465517
Column 9	30	3628500	120950	22294828
Column 10	30	2360500	78683.33	40340230
Column 11	30	2408000	80266.67	33072989
Column 12	30	2354250	78475	45919612

Anova

Source of variation	*SS*	*df*	*MS*	*F*	*P-value*	*F crit*
Between Groups	1.14E^12	11	1.04E^11	2991.254	<0.0001	1.816209
Within Groups	1.21E^10	348	34767511			
Total	1.16E^12	359				

Completely randomized design

```
Data:
========================================================================
 Treatments          1ª Rep.   2ª Rep.   3ª Rep.   4ª Rep.   5ª Rep.
------------------------------------------------------------------------
 Treatment 1         24750.00  20250.00  20750.00  26250.00  24500.00
 Treatment 2         20750.00  27750.00  22250.00  27500.00  24750.00
 Treatment 3         22250.00  24500.00  17500.00  18000.00  26500.00
 Treatment 4         190000.0  166750.0  168000.0  182500.0  184500.0
 Treatment 5         191000.0  195000.0  186250.0  171250.0  176750.0
 Treatment 6         185000.0  176250.0  180250.0  183500.0  181750.0
 Treatment 7         123250.0  112250.0  125750.0  124500.0  111250.0
 Treatment 8         133500.0  119000.0  128000.0  114500.0  128750.0
 Treatment 9         127500.0  124750.0  116750.0  118500.0  128250.0
```

Treatment 10	84000.00	85000.00	85250.00	90000.00	87750.00
Treatment 11	75250.00	82500.00	92250.00	81000.00	83500.00
Treatment 12	84750.00	91500.00	82000.00	74250.00	72750.00

Treatments	6ª Rep.	7ª Rep.	8ª Rep.	9ª Rep.	10ª Rep.
Treatment 1	22750.00	20500.00	25750.00	19750.00	21250.00
Treatment 2	19750.00	26500.00	20750.00	22250.00	21500.00
Treatment 3	26500.00	25250.00	22250.00	19000.00	23750.00
Treatment 4	182750.0	191000.0	170250.0	172500.0	174500.0
Treatment 5	177750.0	173750.0	178000.0	190500.0	184250.0
Treatment 6	179750.0	173750.0	172500.0	175000.0	172000.0
Treatment 7	118000.0	119750.0	128250.0	112000.0	111750.0
Treatment 8	120250.0	112750.0	108000.0	123750.0	119500.0
Treatment 9	126750.0	122000.0	119000.0	118500.0	118750.0
Treatment 10	78250.00	73500.00	77250.00	80500.00	74000.00
Treatment 11	90000.00	89500.00	90750.00	82250.00	79000.00
Treatment 12	80500.00	81000.00	79250.00	86500.00	89250.00

Treatments	11ª Rep.	12ª Rep.	13ª Rep.	14ª Rep.	15ª Rep.
Treatment 1	28750.00	23000.00	26250.00	27000.00	22750.00
Treatment 2	20000.00	21750.00	25500.00	20750.00	21000.00
Treatment 3	15250.00	27000.00	19250.00	28750.00	27000.00
Treatment 4	180250.0	176750.0	171250.0	185000.0	180000.0
Treatment 5	187500.0	183500.0	182250.0	177250.0	183750.0
Treatment 6	182250.0	174500.0	180750.0	170750.0	174750.0
Treatment 7	122250.0	127750.0	112250.0	129250.0	123500.0
Treatment 8	110500.0	120500.0	129750.0	124250.0	132500.0
Treatment 9	129250.0	125500.0	121500.0	115000.0	117000.0
Treatment 10	86000.00	80250.00	69000.00	77000.00	85500.00
Treatment 11	80500.00	78500.00	83250.00	81750.00	82000.00
Treatment 12	78750.00	73000.00	64750.00	69000.00	86500.00

Treatments	16ª Rep.	17ª Rep.	18ª Rep.	19ª Rep.	20ª Rep.
Treatment 1	24750.00	20250.00	21750.00	28250.00	26500.00
Treatment 2	28250.00	26000.00	22250.00	26000.00	24250.00
Treatment 3	24000.00	28750.00	22750.00	22500.00	21500.00
Treatment 4	178250.0	168500.0	172750.0	186500.0	180750.0
Treatment 5	183000.0	167000.0	169750.0	173500.0	174000.0
Treatment 6	172500.0	171000.0	179000.0	182750.0	164250.0
Treatment 7	112000.0	121250.0	122750.0	114500.0	115250.0
Treatment 8	121750.0	126250.0	124500.0	112750.0	128750.0
Treatment 9	124500.0	125250.0	118250.0	112750.0	124500.0
Treatment 10	74000.00	68000.00	70750.00	76250.00	84500.00
Treatment 11	74000.00	79750.00	77000.00	74250.00	81000.00
Treatment 12	81000.00	68250.00	78750.00	74500.00	89250.00

Treatments	21ª Rep.	22ª Rep.	23ª Rep.	24ª Rep.	25ª Rep.
Treatment 1	23250.00	21000.00	26250.00	22250.00	25000.00
Treatment 2	20250.00	19250.00	21500.00	25500.00	25250.00
Treatment 3	21000.00	20750.00	26750.00	24250.00	28500.00
Treatment 4	167500.0	184750.0	165500.0	187500.0	159500.0
Treatment 5	178250.0	180500.0	181750.0	172500.0	185250.0
Treatment 6	182500.0	176500.0	179750.0	181500.0	167500.0

Treatment 7	113750.0	127000.0	131250.0	121000.0	129000.0
Treatment 8	136500.0	133000.0	118250.0	117750.0	126750.0
Treatment 9	125250.0	121500.0	123000.0	113000.0	118250.0
Treatment 10	76750.00	83250.00	70500.00	86250.00	73250.00
Treatment 11	71250.00	74500.00	80750.00	78000.00	71000.00
Treatment 12	73500.00	78500.00	75750.00	84250.00	73000.00

Treatments	26ª Rep.	27ª Rep.	28ª Rep.	29ª Rep.	30ª Rep.
Treatment 1	21500.00	26500.00	24500.00	24750.00	29000.00
Treatment 2	23750.00	25500.00	26500.00	22000.00	22750.00
Treatment 3	26750.00	24000.00	26250.00	26500.00	21000.00
Treatment 4	171750.0	172500.0	174250.0	170250.0	180000.0
Treatment 5	175000.0	170250.0	176000.0	178500.0	172500.0
Treatment 6	185250.0	177250.0	172500.0	171000.0	174250.0
Treatment 7	116000.0	117750.0	118000.0	129500.0	115250.0
Treatment 8	107750.0	111750.0	135000.0	115750.0	118000.0
Treatment 9	123250.0	123750.0	115500.0	117000.0	113750.0
Treatment 10	68750.00	82000.00	82750.00	71500.00	78750.00
Treatment 11	73000.00	88750.00	79000.00	73750.00	80000.00
Treatment 12	83000.00	69500.00	82750.00	73250.00	75250.00

Descriptive treatments statistics :

Treatments	Average	Variance	SD	SEM
Treatment 1	23991.6667	7351221.26	2711.31357	495.015867
Treatment 2	23391.6667	7119324.71	2668.20627	487.145588
Treatment 3	23600.0000	12640517.2	3555.35051	649.115225
Treatment 4	176541.667	64077945.4	8004.87011	1461.48264
Treatment 5	179216.667	47291954.0	6876.91457	1255.54708
Treatment 6	176675.000	28707543.1	5357.94206	978.221909
Treatment 7	120200.000	40928448.3	6397.53455	1168.02466
Treatment 8	122000.000	67465517.2	8213.73954	1499.61681
Treatment 9	120950.000	22294827.6	4721.73989	862.067816
Treatment 10	78683.3333	40340229.9	6351.39590	1159.60094
Treatment 11	80266.6667	33072988.5	5750.91197	1049.96807
Treatment 12	78475.0000	45919612.1	6776.40111	1237.19592

Analysis of Variance

	df	SS	MS	F	P-value
Treatments	11	1.1439E^12	103998466840	2991.25**	< 0.0001
Residual	348	1209909375	34767510.776	-	-
Total	359	1.156082E^12		-	-

General Average..............: 100332.64
Standard deviation...........: 5896.3981
standard error of the mean...: 1076.5301
coefficient of variation.....: 5.8768494

Comparison of means tests

Tukey test

Treatment	Variable 1	
Treatment 5	179216.67	a
Treatment 6	176675.00	a
Treatment 4	176541.67	a
Treatment 8	122000.00	b
Treatment 9	120950.00	b
Treatment 7	120200.00	b
Treatment 11	80266.66	c
Treatment 10	78683.333	c
Treatment 12	78475.000	c
Treatment 1	23991.667	d
Treatment 3	23600.000	d
Treatment 2	23391.667	d

significant difference(5%) = 5009.6171
